# Editorial: Therapeutic potential of smart hydrogel and nanomaterial carriers in neurogenic disease

**DOI:** 10.3389/fnins.2023.1285763

**Published:** 2023-09-13

**Authors:** Kun Lei, Yanyan Jiang, Yi Cao, Xin Wei

**Affiliations:** ^1^School of Medical Technology and Engineering, Henan University of Science and Technology, Luoyang, China; ^2^School of Materials Science and Engineering, Shandong University, Jinan, China; ^3^School of Public Health, University of South China, Hengyang, China; ^4^School of Medicine, University of Nebraska Medical Center, Omaha, NE, United States

**Keywords:** neurogenic disease, delivery carriers, liposomes, nanomaterials, hydrogels, nanocomposites, stem cells, proteins

We are excited to present the Research Topic “*Therapeutic potential of smart hydrogel and nanomaterial carriers in neurogenic disease*”. This Research Topic aims to integrate recent progress in hydrogel-nanomaterials function and utility, specifically the ability as delivery carriers in neurological diseases, including diagnostics, therapeutics, and theranostic applications. Each paper in the Research Topic concentrates on one aspect of neurogenic disease about delivery techniques or characterization methods. The topics covered in this Research Topic are diverse, containing one original research and three reviews-from stimulus-responsive hydrogels and hydrogel scaffolds to nanoparticles, and from drug delivery techniques to diagnosis characterization.

An article from the laboratory of Dr. Shiyu Shu from the Department of Anesthesiology at The Second Affiliated Hospital of Chongqing Medical University, China, developed reactive oxygen species (ROS)-responsive N-hydroxy-N-4-butyl-2-methylphenylformamidine (HET0016) prodrug-loaded liposomes (HPLs) to attenuate neuroinflammation and improve neurological deficit in a rat model of juvenile traumatic brain injury (TBI) (Qin et al.). Specifically, the authors first designed a ROS-responsive HET0016 prodrug, consisting of a thioketal link between HET0016 and stearyl alcohol (HET-TK-SA), and then used the nanoprodrug strategy to successfully synthesize HPLs to facilitate the application of HET0016 in protection from TBI. The obtained HPLs showed spherical shape, a narrow particle size distribution and good stability with size of 127.8 nm, a zeta potential of −28.8 mv. Compared with HET0016, HPLs can effectively inhibit inflammation and improve neuronal degeneration, further leading to lesion volume reduction, upgrading behavioral task performance, and ameliorating the degree of TBI impairment.

A review paper from the laboratory of Dr. Fengshou Zhang from the College of Medical Technology and Engineering at Henan University of Science and Technology, China, provides an conclusion of recent research progress in functionalized hydrogels for nerve (the central and peripheral nervous systems) injury repair, highlighting the design differences among various materials and future research directions. These functionalized hydrogels for neural injury repair include dopamine-functionalized-, conductive, extracellular vesicles functionalized-, nanomaterials functionalized-hydrogels, *etc*. The authors conclude and compare various synthesized strategies of functionalized hydrogels and their biological performances in neural injury repairing (Zhao et al.).

A hydrogel scaffold in the treatment of spinal cord injury (SCI) is described by Dr. Ziyi Li and Hongfu Wu from Dongguan Key Laboratory of Stem Cell and Regenerative Tissue Engineering, The First Dongguan Affiliated Hospital, Guangdong Medical University, China (Cai et al.). In this paper, the authors introduce several typical composite hydrogel scaffolds and review the research progress for SCI to provide a reference for the clinical application of hydrogel therapy for SCI. The authors demonstrate the fabricated strategies of composite hydrogel scaffolds (natural-synthetic-, nanoparticle-, nanotube-, microsphere-composite hydrogel scaffolds) and compare their advantages and disadvantages of physical, chemical, biological properties. Then, several stimuli-responsive hydrogels, for example temperature-, light-, pH-, and electricity-responsive hydrogels, are introduced to facilitate their applications in tissue engineering, cell and drug delivery, and regulation of the tissue environment to promote innate tissue repair.

Lastly, a review paper from the laboratory of Dr. Zhigang Hu from the College of Medical Technology and Engineering, Henan University of Science and Technology, China, concluded the relevant papers published from 2010 to 2022, using diffusion magnetic resonance imaging (MRI) technique for white matter disconnections associated with behavioral disturbances in mild cognitive impairment (MCI). It can be considered that fibers connected to the hippocampus and temporal lobe are associated with cognition decline in MCI, and fibers connected to the thalamus are associated with both cognition and affection abnormality. The review summarized the correspondence between white matter disconnections and behavioral disturbances such as cognition and affection, providing a theoretical basis for the future diagnosis and treatment of Alzheimer's disease (AD) (Zhou et al.).

The papers in this Research Topic represent a recent progress in hydrogel-nanomaterials function and utility as delivery carriers, and delivery techniques or characterization methods in neurological diseases. It is supposed that this Research Topic will provide beneficial inspiration in realizing the therapeutic potential of smart hydrogel and nanomaterial carriers and diagnostic method innovation for neurogenic disease.

## Author contributions

KL: Conceptualization, Writing—original draft. YJ: Writing—review and editing, Investigation. YC: Writing—review and editing, Supervision. XW: Formal analysis, Writing—review and editing.

